# Application of the AI-Based Framework for Analyzing the Dynamics of Persistent Organic Pollutants (POPs) in Human Breast Milk

**DOI:** 10.3390/toxics13080631

**Published:** 2025-07-27

**Authors:** Gordana Jovanović, Timea Bezdan, Snježana Herceg Romanić, Marijana Matek Sarić, Martina Biošić, Gordana Mendaš, Andreja Stojić, Mirjana Perišić

**Affiliations:** 1 Institute of Physics Belgrade, a National Institute of the Republic of Serbia, Pregrevica 118, 11080 Belgrade, Serbia; gordana.jovanovic@ipb.ac.rs (G.J.); andreja.stojic@ipb.ac.rs (A.S.); mirjana.perisic@ipb.ac.rs (M.P.); 2 Faculty of Informatics and Computing, Singidunum University, Danijelova 32, 11000 Belgrade, Serbia; tbezdan@singidunum.ac.rs; 3 Institute for Medical Research and Occupational Health, Ksaverska Cesta 2, P.O. Box 291, 10001 Zagreb, Croatia; mbiosic@imi.hr (M.B.); gmendas@imi.hr (G.M.); 4 Department of Health Studies, University of Zadar, Splitska 1, 23000 Zadar, Croatia; marsaric@unizd.hr; 5 Environment and Sustainable Development Studies, Singidunum University, Danijelova 32, 11000 Belgrade, Serbia

**Keywords:** breast milk, polychlorinated biphenyls (PCBs), organochlorine pesticides (OCPs), machine learning, metaheuristics, explainable artificial intelligence

## Abstract

Human milk has been used for over 70 years to monitor pollutants such as polychlorinated biphenyls (PCBs) and organochlorine pesticides (OCPs). Despite the growing body of data, our understanding of the pollutant exposome, particularly co-exposure patterns and their interactions, remains limited. Artificial intelligence (AI) offers considerable potential to enhance biomonitoring efforts through advanced data modelling, yet its application to pollutant dynamics in complex biological matrices such as human milk remains underutilized. This study applied an AI-based framework, integrating machine learning, metaheuristic hyperparameter optimization, explainable AI, and postprocessing, to analyze PCB-170 levels in breast milk samples from 186 mothers in Zadar, Croatia. Among 24 analyzed POPs, the most influential predictors of PCB-170 concentrations were hexa- and hepta-chlorinated PCBs (PCB-180, -153, and -138), alongside *p*,*p’*-DDE. Maternal age and other POPs exhibited negligible global influence. SHAP-based interaction analysis revealed pronounced co-behavior among highly chlorinated congeners, especially PCB-138–PCB-153, PCB-138–PCB-180, and PCB-180–PCB-153. These findings highlight the importance of examining pollutant interactions rather than individual contributions alone. They also advocate for the revision of current monitoring strategies to prioritize multi-pollutant assessment and focus on toxicologically relevant PCB groups, improving risk evaluation in real-world exposure scenarios.

## 1. Introduction

Human milk is widely regarded as the gold standard for infant nutrition, providing essential macronutrients, vitamins, and minerals. In addition to its nutritional value, breast milk contains a wide range of bioactive and immunological components, such as immunoglobulins, lymphocytes, interleukins, lactoferrin, and non-digestible oligosaccharides, that help protect neonates against intestinal infections and stimulate immune development [1]. The composition of breast milk is dynamic and influenced by factors such as infant age, gestational maturity, and nutritional status. However, breast milk can also serve as a pathway for environmental contaminants, potentially exposing infants to harmful chemicals during a highly sensitive developmental stage.

Owing to its dual role as both a source of infant nutrition and an indicator of maternal body burden, human milk has served as a key matrix for biomonitoring, with global studies over the past 70 years tracking contaminants such as persistent organic pollutants (POPs) and toxic metals. DDT, the first pollutant detected in human milk in 1951, remains nearly ubiquitous, with subsequent attention focused on lipophilic xenobiotics such as polychlorinated biphenyls (PCBs), organochlorine pesticides (OCPs), dioxins, organophosphate pesticides, bisphenols, and polycyclic aromatic hydrocarbons, which accumulate in maternal adipose tissue [2]. Following the Stockholm Convention, biomonitoring efforts expanded with harmonized sampling protocols and national breast milk pools, while studies worldwide, including those in Colombia [3], Croatia [4], the Czech Republic [5], Norway [6], and Mexico [7], have continued to reveal POP temporal trends. In addition, pooled samples from 90 countries for 29 POPs revealed overall declining trends, with most reductions occurring after 2000, though significant differences between earlier 5-year periods dating back to the mid-1980s diminished over time, and levels generally plateaued in samples from 2015 to 2019 [8]. Despite growing data, knowledge of co-exposure patterns and pollutant interactions remains limited.

The exposome, a relatively recent concept, encompasses the totality of environmental exposures and associated biological responses across an individual’s lifespan, including those from diet, behavior, endogenous processes, and the external environment [9]. This approach represents a shift from traditional exposure science focused on individual chemicals toward a more holistic view of complex exposure mixtures and their health and environmental impacts. Characterizing chemical mixtures and co-contamination patterns is essential to understanding the exposome, enhancing source attribution, and ultimately improving the prioritization of chemicals in risk assessment [2].

The 2024 Nobel Prizes in physics and chemistry, awarded for foundational advances in neural networks and protein structure prediction, highlight the growing role of artificial intelligence (AI) in transforming science and engineering. These honors underscore that AI is becoming a core component of modern scientific inquiry, not just a domain-specific innovation [10]. Despite notable successes in healthcare, climate research, air quality monitoring, and energy management, the adoption of artificial intelligence in environmental science has been met with resistance. Key challenges include the scarcity of high-quality data; the prevalence of missing or noisy inputs; and the “black box” nature of many machine learning (ML) models, which often lack interpretability and mechanistic transparency. These factors have contributed to skepticism among environmental professionals. Nevertheless, AI holds a significant promise for transforming environmental science by enhancing data processing, improving the understanding of pollutant dynamics across environmental matrices, and enabling more accurate forecasting and decision making. To unlock this potential, it is essential to combine domain expertise with robust AI methodologies and enhance model transparency through explainable AI (XAI). As the field matures, ongoing research continues to address both the limitations and the environmental impact of AI itself, ensuring that its application supports sustainable and scientifically grounded outcomes [11].

While AI adoption in environmental health has faced challenges, its application in the breastfeeding domain is increasingly evident. For example, Souza et al. (2023) applied ML to study the co-exposure of lactating Brazilian women and their infants to organic contaminants such as polycyclic aromatic hydrocarbons (PAHs) and toxic metals [12]. Despite using 16 ML algorithms, no association was found between these pollutants and oxidative stress biomarker (8-hydroxydeoxyguanosine) urinary levels, highlighting the novelty and robustness of the approach in revealing non-obvious patterns that traditional models missed. Recently, Huang et al. (2025) introduced an explainable AI framework for assessing chemical risks of xenobiotics such as POPs in breastfeeding infants [13]. Their optimized model, combining ensemble resampling, genetic algorithms, and Shapley additive explanations (SHAP) for interpretability, achieved over 86% accuracy in external validation. This approach not only improves predictive performance but also identifies key molecular features driving chemical transfer into breast milk, offering a powerful tool for targeted risk assessment and evidence-based public health strategies.

Our previous studies employed ML to investigate PCB and OCP levels in human milk in relation to maternal factors such as age and parity. Guided Regularized Random Forest (GRRF) modelling identified key predictors of POP levels and revealed complex non-linear relationships [14], while Extreme Gradient Boosting (XGBoost) and SHAP analyses highlighted PCB-170 and PCB-153 as major drivers of PCB-138 behavior. Building on this foundation, the present study focuses on the co-occurrence and associations of PCB-170 with other congeners. In contrast to commonly monitored indicator congeners such as PCB-28, PCB-52, PCB-101, PCB-153, PCB-138, and PCB-180, which are frequently reported due to their high prevalence, long biological half-lives, and regulatory importance, PCB-170, a non-dioxin-like congener, is less frequently detected and typically occurs at lower concentrations. Nevertheless, it remains toxicologically relevant due to its persistence and potential for bioaccumulation. While PCB-170 is not usually prioritized in routine exposure assessments, in this study, it was selected as the predicted variable to explore the feasibility of using ML and explainable AI to leverage relationships between commonly and less commonly detected PCB congeners. Specifically, our objective was to investigate whether the concentrations of more prevalent congeners could be used to predict and better understand the behavior and co-exposure patterns of less frequently detected PCBs such as PCB-170. In this study, we utilized an AI framework [15,16] to investigate factors influencing the co-distribution of PCB-170 in breast milk and to assess its potential as a marker of overall PCB exposure. The framework, representing a new conceptual approach in environmental data analysis, is designed as a modular and flexible analytical environment. It integrates ensemble regression algorithms known for their predictive strength, hyperparameter tuning using a diverse set of metaheuristic optimization algorithms, explainable AI methods enabling transparent interpretation of model predictions, and interactive visualizations for deeper exploration. The obtained results highlight its broader potential for addressing complex environmental health challenges.

## 2. Materials and Methods

### 2.1. Sample Collection

For the purposes of this study, analysis was conducted on a total of 186 breast milk samples selected from our previous research [14,17]. The samples were collected during two periods: from 2011 to 2014 and from January 2018 to March 2019. The study involved healthy mothers from the general population of the Zadar region, Croatia, aged 19 to 41 years and with one, two, or a maximum of three deliveries. Zadar does not have significant industrial pollution sources and is not categorized among highly polluted areas. Its selection was based on practical considerations, including ease of participant recruitment and sample collection. In the absence of major localized pollution, the cohort represents exposure conditions appropriate for evaluating general population exposure to POPs.

Breast milk was manually expressed 2 to 38 weeks postpartum into pre-cleaned glass bottles provided by research staff. Samples were stored at −20 °C until analysis. For freeze-dried preparations (collected between 2018 and 2019), 50 mL of liquid milk was used to obtain 5 g of freeze-dried milk. Frozen samples were stored at −80 °C and freeze-dried in a Labconco FreeZone Benchtop freeze dryer at −50 °C for 48 h. The resulting dried samples were stored at room temperature in a dark desiccator.

Participants were recruited through the University of Zadar, Department of Health Studies, in cooperation with the Zadar County Health Center, Community and Primary Health Care Division (visiting nurses). Recruitment aimed to reflect a diverse population in terms of age and childbirth history, while considering lifestyle factors such as smoking habits and potential exposure to organic pollutants through occupation or residential environment.

Mothers voluntarily participated after receiving detailed information about the study’s goals and relevance. Written informed consent was obtained prior to inclusion. The ethical conduct of the study was approved by the Ethics Committee of the Zadar County Health Center under reference numbers 01–745/2011, 01–405/2014, and 01–5471/2017. All personal and clinical data, as well as biological samples, were coded to ensure participant anonymity and used solely for research purposes.

Participants confirmed that they had no history of accidental or occupational exposure to the persistent organic pollutants (POPs) under investigation, including PCBs and OCPs. Detailed sampling procedures and additional data on maternal lifestyle and habits have been described previously [4,14,17].

### 2.2. Chemical Analysis

In this study, we analyzed 17 PCBs including 6 indicator congeners (PCB-28, PCB-52, PCB-101, PCB-138, PCB-153, and PCB-180), 8 mono-ortho congeners (PCB-105, PCB-114, PCB-118, PCB-123, PCB-156, PCB-157, PCB-167, and PCB-189), and 3 additional congeners (PCB-60, PCB-74, and PCB-170). The analysis also included OCPs such as hexachlorobenzene (HCB); the α-, β-, and ɣ-isomers of hexachlorocyclohexane (α-, β-, and ɣ-HCH, with the ɣ-isomer commonly known as lindane); and 1,1,1-trichloro-2,2-di(4-chlorophenyl)ethane (*p*,*p’*-DDT), 1,1-dichloro-2,2-di(4-chlorophenyl)ethane (*p*,*p’*-DDD), and 1,1-dichloro-2,2-di(4-chlorophenyl)ethylene (*p*,*p’*-DDE). The analytical procedure adhered to established protocols for extraction, purification, and quantification of PCBs, as described in detail in previous studies [4,17].

Briefly, two 5 g subsamples of each milk sample were extracted twice using a 1:1 mixture of chloroform and methanol (17 mL and 10 mL, respectively). Chloroform layers were separated and dried under nitrogen, followed by milk fat weighing and dissolution in n-hexane. Subsequently, clean-up involved sulfuric acid (5 mL) treatment and adsorption chromatography on a multilayer silica column using 4% diethyl ether in n-hexane. The fractionation was performed by ENVI-Carb SPE tubes (3 mL, 0.25 g; Supelco, Bellefonte, PA, USA), while elution was performed with n-hexane/toluene (99:1). Final extracts were evaporated to dryness, re-dissolved in n-hexane, and analyzed [4,17].

For freeze-dried samples, 5 g of milk powder was mixed with 5 mL methanol and diatomaceous earth, then transferred to 60 mL stainless steel Accelerated Solvent Extraction (ASE) cells. Extraction was performed at 125 °C and 1500 psi, with a static time of 5 min over three cycles, using n-hexane as the solvent and a 70% flush volume. Extracts were evaporated to dryness using a GeneVac Rocket evaporator and reconstituted in 2 mL n-hexane. One aliquot (1 mL) was used for PCB. The extract was purified with two successive 5 mL sulphuric acid treatments, followed by centrifugation (10 min at 4500 rpm). The n-hexane layer was separated, evaporated under nitrogen, and reconstituted in 1 mL n-hexane [17].

Two instrumental analysis methods were employed, depending on the year the samples were collected. The first involved quantification of PCBs using high-resolution gas chromatography coupled with electron capture detection (HRGC-ECD) on a CLARUS 500 system (PerkinElmer) [4,17]. Two capillary columns (Restek, Bellefonte, PA, USA) were used: Rtx-5 (60 m × 0.25 mm i.d., 0.25 μm film) and Rtx-1701 (30 m × 0.25 mm i.d., 0.25 μm film). The oven temperature was initially ramped from 100 °C to 110 °C at a rate of 4 °C min^−1^ with a 5 min hold at 110 °C, followed by an increase from 110 °C to 240 °C at 15 °C min^−1^, with a final hold of 50 min at 240 °C. Injector and detector temperatures were 250 °C and 270 °C, respectively. Nitrogen was used as the carrier gas.

The second instrumental analysis was carried out using an Agilent 7890B gas chromatograph equipped with dual micro-electron capture detectors (μECDs) and two capillary columns: HP-5 and SDB-1701 (both 30 m × 0.25 mm, 0.25 μm film thickness) [17]. The oven temperature program was as follows: an initial hold at 90 °C for 1 min, followed by a ramp to 180 °C at 30 °C min^−1^ with a 1 min hold, then increased to 240 °C at 2 °C min^−1^ and held for 20 min, followed by a final ramp to 260 °C at 5 °C min^−1^ with an 8 min hold. Helium was used as the carrier gas at a flow rate of 1.2 mL min^−1^. The injection volume was 1 μL, with injector and detector temperatures set at 270 °C and 300 °C, respectively.

### 2.3. Quality Assurance and Quality Control

All samples were analyzed in duplicate using both instrumental protocols and chromatographic columns. Only compounds consistently identified on both columns were quantified, and their concentrations were reported as the average of the two measurements. Five calibration standards, with concentrations ranging from 0.1 to 5.0 ng mL^−1^, were prepared and demonstrated good linearity across this range.

To account for variations in breast milk composition over time and extended sampling time (from 2 to 38 weeks), all PCB and OCP concentrations were adjusted for lipid content. This standardization reflects the lipophilic nature of these compounds, which accumulate in adipose tissue and are excreted slowly [18].

The limits of determination (LOD) were calculated as the average of ten measurements (N = 10), based on a signal-to-noise ratio of 3:1 or higher and compound recovery. The LOD values ranged from 0.01 to 0.6 ng g^−1^ milk fat, while recoveries, determined using spiked real samples (n = 10), ranged from 58% to 105%. The relative standard deviations (RSD) were between 6% and 22%.

### 2.4. Data Analysis

Data analysis, targeting PCB-170 concentrations, was carried out using the modular and automated AI-driven framework that sequentially integrates advanced ML, metaheuristic parameter optimization, explainable AI methods, and postprocessing of the obtained results. In brief, PCB-170 concentrations were modeled using a suite of ML algorithms. The top three performing models were further optimized via metaheuristic techniques. The best-performing model was interpreted using both Shapley additive global importance (SAGE) and SHAP explainability methods. Finally, SHAP values were subjected to clustering analysis, yielding clusters that reflect higher-order relationships and group-specific patterns among predictors. At its core, the framework incorporates six ensemble regression algorithms: AdaBoost, LightGBM, XGBoost, ExtraTrees, Gradient Boosting, and Histogram-Based Gradient Boosting, implemented via scikit-learn (v1.4), LightGBM (v4.5.0), and XGBoost (v2.1.3) [19,20,21]. These were selected for their ability to capture complex nonlinear patterns and their resilience against overfitting. AdaBoost reduces bias by iteratively emphasizing misclassified instances, while LightGBM enhances efficiency and scalability. XGBoost is known for its robust regularization, parallel processing capabilities, and flexible loss functions. ExtraTrees introduces randomness in feature splits to reduce variance and improve performance in high-dimensional spaces. Gradient Boosting incrementally refines predictions by correcting previous errors, and its histogram-based variant accelerates training by grouping features into discrete bins.

All six algorithms were applied to the dataset in order to evaluate their baseline performance in predicting PCB-170 concentrations. Model performance was assessed using fivefold cross-validation, ensuring generalizability and minimizing overfitting. Based on key evaluation metrics, R-squared (R^2^), mean absolute error (MAE), and mean squared error (MSE), the three top-performing models were selected for further refinement. To enhance their predictive accuracy, hyperparameter optimization was conducted using two metaheuristic algorithms: Harris Hawks Optimization (HHO) and the Sine Cosine Algorithm (SCA) [22,23], implemented through the Mealpy library (v3.0.1). HHO mimics the cooperative hunting behavior of hawks to achieve a balance between exploration and exploitation, while SCA utilizes trigonometric operators to effectively traverse complex and non-convex search spaces. Both algorithms proved effective in fine-tuning model parameters, resulting in notable improvements in prediction accuracy. Among the optimized models, the one achieving the highest R2 value was selected as the final model, offering the best combination of predictive power, generalizability, and robustness for estimating PCB-170 concentrations.

Beyond optimizing predictive accuracy, this study emphasized model transparency by incorporating XAI methods to ensure interpretability. SHAP quantifies the contribution of each feature to individual predictions, providing local, instance-level insight [24], while SAGE aggregates these contributions across the entire dataset to evaluate each feature’s overall impact on model performance [25]. To further enhance interpretability, we introduced derivative metrics. One such metric, relative SHAP, captures each feature’s proportion of the total attribution for a given prediction, enabling more intuitive and normalized comparisons across features. SHAP interaction values extend the original SHAP concept by estimating how pairs of features jointly influence model outputs. Specifically, they measure how the combined contribution of two features differs from the sum of their individual effects, thus capturing second-order interactions. Such patterns may suggest either data-level correlations or model-learned dependencies that follow a consistent, possibly linear, interaction structure.

To explore higher-order relationships and group-specific patterns among predictors, the postprocessing of the SHAP values using a cluster analysis was conducted. Dimensionality reduction was performed using Pairwise Controlled Manifold Approximation (PCMA) [26], followed by unsupervised clustering with Hierarchical Density-Based Spatial Clustering of Applications with Noise (HDBSCAN) [27,28]. This approach enabled the identification of feature interaction patterns, potential outliers, and distinct subgroups within the data, based on model-inferred behavior.

## 3. Results and Discussion

Table S1 (Supplementary Material) summarizes the descriptive statistics parameters, while Figure S1 shows the distribution, central tendency, and variability through violin plots for the compounds identified as relevant for further analysis, as discussed below. In general, the violin plots show the presence of a long, narrow upper tail, which indicates the presence of a few high outliers. PCB levels ranged from 0.01 ng g^−1^ to 32.5 ng g^−1^, whereas OCP concentrations varied between 0.01 ng g^−1^ and 92.6 ng g^−1^ with the most prevalent compounds, in decreasing order, being *p*,*p’*-DDE, PCB-153, PCB-138, PCB-180, β-HCH, PCB-118, γ-HCH, HCB, PCB-156, and PCB-170. PCB-28, PCB-105, and PCB-60 were among the least abundant compounds. Detailed description of the dataset used in this study, along with comparisons to other studies, have been previously documented [4,17].

As indicated by Pearson correlation coefficients (r > 0.90), strong linear relationships were observed between PCB-170 and PCB-138, PCB-153, and PCB-180, as well as between PCB-180 with PCB-153 (Figure S2). These high correlation values suggest a potentially common origin and similar metabolic behavior within the maternal organism, including similar dynamics of excretion into breast milk. In addition, moderate correlations (r = 0.60–0.70) were observed between *p*,*p’*-DDE; the major metabolite of *p*,*p’*-DDT; and several PCBs: PCB-170, PCB-138, PCB-153, PCB-180, and PCB-118. While these moderate correlations do not indicate direct interrelations or identical biochemical behavior, they suggest possible co-occurrence in environmental and biological matrices, likely attributable to their shared persistence and comparable bioaccumulation and elimination profiles in the human body.

### 3.1. Model-Derived Findings

The ExtraTrees model optimized via the Sine Cosine Algorithm emerged as the most accurate, achieving an R2 of 0.91, RMSE of 0.74, and MAPE of 0.32 (Table S2, Figure S3, Supplementary Material). These results reflect strong predictive performance and effective modeling of variance in PCB-170 concentrations. Among the 24 POPs analyzed, PCB-180 (35%), PCB-153 (31%), and PCB-138 (20%) exhibited the highest average contributions to PCB-170 prediction, followed by PCB-156 (6%) and *p*,*p’*-DDE (1%), as indicated by their relative global importance values exceeding 1%, quantified using SAGE (Figure S4, Supplementary Material). Additionally, the strongest correlations between the obtained SHAP values were observed among these key pollutant pairs (Figure S5, Supplementary Material), suggesting consistent model-learned associations.

The compounds PCB-180, PCB-153, PCB-138, and PCB-170 share several key characteristics that help explain their prominence in environmental matrices such as breast milk. As hexa- and hepta-chlorinated biphenyls, they are commonly found together in commercial PCB mixtures and exhibit highly similar molecular structures. These structural features contribute to their pronounced environmental persistence and their ability to bioaccumulation and biomagnification through the food chain. Recent research by Huang et al. (2025) identified nine molecular fragments as potential structural alerts for chemicals with high transfer potential into breast milk, using the milk-to-plasma concentration ratio as the key indicator [13]. Values above 1–5 on this scale suggest significant chemical accumulation and, consequently, increased infant exposure [29,30]. Among the 83 chemicals classified as high risk, 3 of the identified fragments were particularly prevalent, with the 1,3-dichlorobenzene substructure appearing in 62 of them. This fragment, frequently found in organochlorine compounds such as highly chlorinated PCBs and *p*,*p’*-DDT metabolites, is associated with increased lipophilicity and metabolic stability. These properties enhance membrane permeability and retention in lipid-rich tissues like breast milk while reducing metabolic breakdown. For instance, both PCBs and *p*,*p’*-DDT have estimated half-lives of approximately six months in human milk [13], further underscoring their capacity for long-term exposure through lactation. This long-term exposure is particularly concerning given the well-documented adverse health effects associated with PCBs, HCB, HCH, *p*,*p’*-DDT, and its metabolite *p*,*p’*-DDE. Due to their lipophilic nature and ability to bioaccumulate in fatty tissues, these compounds readily concentrate in human milk and exert endocrine-disrupting effects [31]. While HCB, HCH, and *p*,*p’*-DDT are classified as probable human carcinogens (IARC Group 2B), PCBs are of particular concern, having been classified as Group 1 carcinogens by IARC [32]. PCB congeners have been linked to increased risks of liver, breast, and lymphatic cancers, as well as immunotoxic and hepatotoxic effects resulting from oxidative stress and enzymatic disruption [33]. They also function as endocrine disruptors, posing heightened risks to children, who are especially vulnerable during periods of rapid hormonal development such as infancy and puberty. Prenatal exposure to PCBs has been associated with adverse neurodevelopmental outcomes, including reduced IQ, attention deficits, and behavioral problems [34]. These findings have been further substantiated and expanded in recent studies [35].

Maternal age had minimal influence (0.07%) on the prediction of PCB-170 levels, while compounds like HCB, ɣ-HCH, PCB-74, PCB-123, PCB-157, and *p*,*p’*-DDD along with the birth-related factor (first, second, and third delivery) also showed negligible impact (Figure S4, Supplementary Material). Although child delivery generally reduces maternal POP levels through breastfeeding, maternal age has been linked to higher POP concentrations. A Norwegian study reported a positive correlation between age and POPs, as well as a negative association with parity [36]. However, a recent large-scale Czech study found that maternal diet before and during pregnancy significantly affected PCB levels, while age and body weight had no notable effect [37]. These results align with our previous study, in which GRRF revealed mutual associations among PCB congeners, but not with maternal factors such as age and parity [14].

#### 3.1.1. Impact of Individual Congeners on PCB-170 Prediction

To reveal latent structure in the distribution of SHAP values, clustering was applied, resulting in ten distinct groups (C0–C9) (Table 1). The largest cluster, C8, encompassed 18.28% of instances and was characterized by low PCB-170 concentrations and negative mean SHAP impacts, suggesting a marginal or suppressive effect on the predicted outcome. In contrast, C2 exhibited the highest average concentration of PCB-170 (7.99 ng g^−1)^ and the strongest positive SHAP influence, both in raw and normalized terms, indicating a dominant contribution to positive model predictions within this group. Clusters such as C1 and C3 also showed substantial positive attributions, though with lower exposure levels, suggesting non-linear or threshold effects.

Negative mean impacts observed in C5–C7 reflect patterns where PCB-170 presence was associated with reduced prediction scores. Notably, C-1 contains 13.44% of data points that were not assigned to any cluster, likely due to their distinct SHAP attribution profiles or insufficient similarity to other groups. These findings highlight the heterogeneity of feature effects across the sample and underscore the value of clustering SHAP outputs in uncovering meaningful substructures in model interpretation.

**Figure 1:**
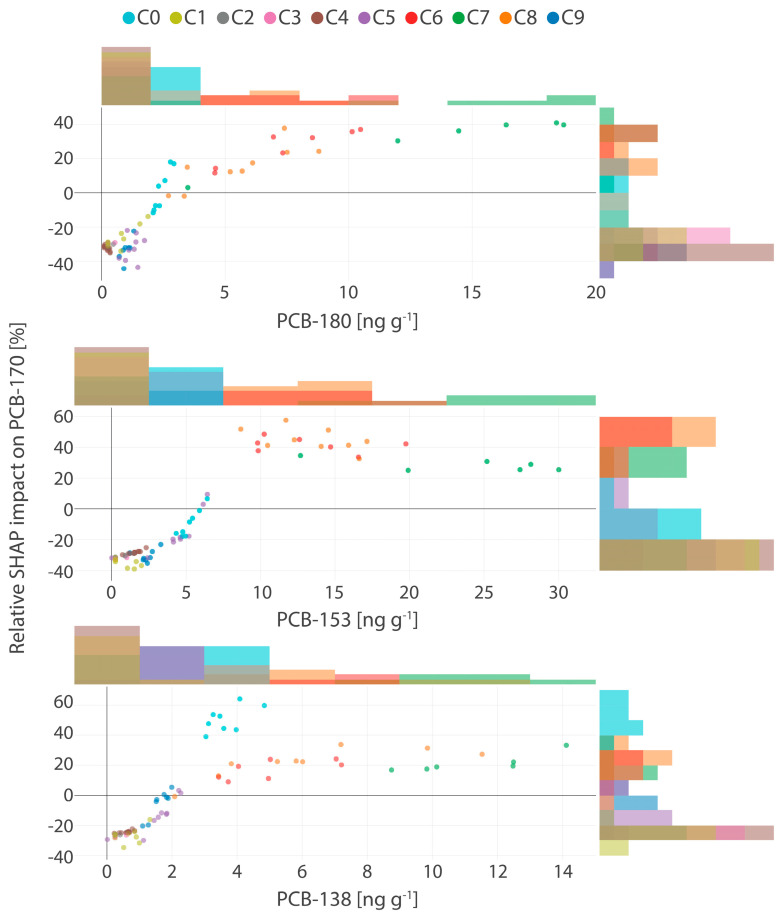
Relative SHAP impacts of PCB-180, PCB-153, and PCB-138 on the PCB-170 predictions in human milk samples.

To further investigate the cluster-specific patterns identified in the SHAP-based analysis, SHAP dependence plots were employed to visualize the individual and relative contributions of PCB-180, PCB-153, and PCB-138, the most influential predictors of PCB-170 concentrations (Figure 1). In these plots, each point represents an individual sample, colored according to its assigned cluster, facilitating the detection of trends across concentration ranges and within cluster-specific contexts.

Across all three predictors, a clear monotonic trend emerged: higher concentrations were generally associated with increased SHAP contributions to PCB-170 predictions. Notably, PCB-138 and PCB-153 exhibited the most pronounced impacts, with relative SHAP contributions reaching up to 60%, particularly in the mid-to-high concentration intervals. For PCB-138 (3.04–14.11 ng g^−1^), SHAP impacts clustered into four distinct groups (C1, C2, C3, and C9), while PCB-153 (8.67–30.01 ng g^−1^) formed three well-defined clusters (C1, C2, and C3), each corresponding to a high positive influence on predicted PCB-170 levels. PCB-180 displayed a more nuanced pattern, with a transition from negative to positive SHAP values across its concentration range (3–18.7 ng g^−1^). In its upper range, C1, C2, C3, and C9 emerged with relative SHAP contributions reaching up to 40%, indicating a strong enhancing effect on predictions. Conversely, at lower concentrations, all three predictors showed stable or marginal influence, with greater variability introduced at higher levels, most prominently for PCB-153. Interestingly, at low exposure levels, these same predictors were associated with negative SHAP contributions, suggesting a suppressive effect on PCB-170 predictions. For instance, PCB-180 in the range of 0.25–1.85 ng g^−1^ (C0, C4–C8) showed negative SHAP values up to –32%. Similarly, PCB-153 (0.01–5.9 ng g^−1^; C0, C4–C9) and PCB-138 (0.51–2.27 ng g^−1^; C0, C4–C8) displayed relative SHAP values as low as –38% and –30%, respectively.

These patterns suggest potential co-behavior and shared exposure pathways among higher-chlorinated congeners, possibly reflecting common sources, similar bioaccumulation dynamics, or overlapping toxicokinetic processing. The concentration-dependent shifts in SHAP contributions may also reflect threshold effects, nonlinear interactions, or individual variability in exposure and elimination. Elucidating these mechanisms will require further investigation using larger datasets and the inclusion of additional exposure-related covariates.

#### 3.1.2. Interaction Effects Between Co-Pollutants on PCB-170 Prediction

To identify variables that not only influence the prediction of PCB-170 individually but also exhibit joint effects beyond their separate contributions, a SHAP interaction matrix was constructed (Figure S6, Supplementary Material). This matrix quantifies how pairs of co-pollutants jointly affect model predictions, with interaction values highlighting mutual influences, particularly among highly chlorinated congeners such as PCB-138–PCB-153, PCB-138–PCB-180, and PCB-180–PCB-153.

**Figure 2:**
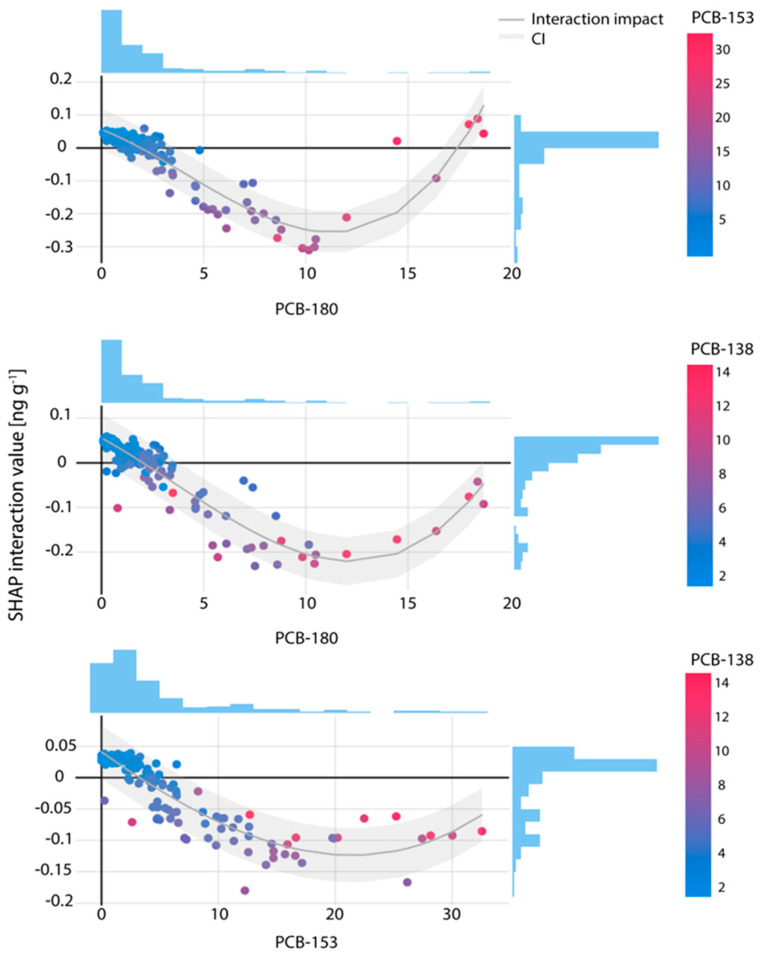
SHAP interaction effects of co-pollutant pairs: PCB-180–PCB-153 (**top**), PCB-180–PCB-138 (**middle**) and PCB-153–PCB-138 (**bottom**), on the PCB-170 predictions in human breast milk; pollutant concentrations are shown on the x-axis and expressed in ng g^−1^ samples.

For the PCB-180–PCB-153 pair, positive SHAP interaction values were observed in both low- and high-concentration ranges: when PCB-180 ranged from 0.09 to 3 ng g^−1^ and PCB-153 from 1.1 to 5.4 ng g^−1^, and again when PCB-180 approached 18 ng g^−1^ and PCB-153 ranged from 30 to 32 ng g^−1^ (Figure 2, top panel). These interactions suggest consistent co-behavior, where increases in one congener’s SHAP value are accompanied by increases in the other, indicating possible shared sources or similar metabolic and environmental pathways.

In contrast, negative SHAP interaction values appeared in a smaller subset of the data, primarily at mid-to-high concentrations where PCB-180 values are between 3.3 and 16 ng g^−1^ and PCB-153 between 12 and 20 ng g^−1^. These negative values imply that the combined predictive effect of the two congeners is less than additive, possibly due to interference or overlapping biological mechanisms.

Such non-additive interactions may reflect non-linear effects or biological saturation, where the presence of one pollutant (e.g., PCB-180) elicits a near-maximal physiological response, limiting the incremental impact of the second (e.g., PCB-153). This plateau effect could explain why further increases in concentration do not enhance prediction and may even reduce the overall contribution due to mutual interference.

An interaction effect on PCB-170 prediction was also evident for the co-pollutant pair PCB-138–PCB-180, as illustrated in the middle panel of Figure 2. In the lower concentration ranges, where PCB-180 concentrations are in range 0.1–2.92 ng g^−1^ and PCB-138 in range 0.1–4.15 ng g^−1^, SHAP interaction values were consistently positive, reaching up to 0.06. This suggests that in this range, the combined presence of these two congeners contribute positively and proportionally to the predicted PCB-170 levels.

In contrast, at higher concentrations of PCB-180 between 3.46 and 18.7 ng g^−1^ and PCB-138 between 2.08 and 14.46 ng g^−1^, the interaction effect shifted toward negative values, with SHAP contributions reaching as low as –0.23. These findings indicate that at elevated levels, the combined influence of these two pollutants is no longer additive and may even suppress the predicted presence of PCB-170, possibly due to metabolic competition or biological saturation.

Notably, in the transition zone with PCB-180 concentrations between 2.92 and 3.46 ng g^−1^, SHAP interaction values ranged from –0.053 to 0.4, exhibiting both positive and negative effects. This variability suggests a threshold-like behavior in the interaction dynamics, where small shifts in concentration may lead to non-linear changes in model output, potentially reflecting inter-individual differences in exposure or toxicokinetic responses.

Interaction analysis between PCB-138 and PCB-153 revealed a more moderate influence on PCB-170 prediction compared to previously examined congener pairs, as shown in the bottom panel of Figure 2. At low concentration levels of PCB-153 (0.01–2 ng g^−1^) and PCB-138 (0.01–1.18 ng g^−1^), SHAP interaction values were consistently positive, though modest, falling within the range of 0.02 to 0.04. These values suggest a limited but coherent co-contribution to the predicted PCB-170 levels in low-exposure conditions.

In contrast, at higher concentrations of PCB-153 (6.57–32.54 ng g^−1^) and PCB-138 (1.84–14.11 ng g^−1^), interaction values turned negative, reaching as low as –0.18. This negative shift indicates that the joint presence of both congeners at elevated levels may attenuate their individual contributions to the model output, possibly due to saturation effects or competitive interactions.

In the intermediate concentration range, PCB-153 between 2 and 6.43 ng g^−1^ and PCB-138 between 0.01 and 3.12 ng g^−1^, SHAP interaction values varied between –0.038 and 0.039, showing a mix of positive and negative effects. This transitional behavior further supports the presence of non-linear dynamics in the interaction between these two congeners.

Overall, PCBs were historically produced as complex mixtures containing multiple congeners, with compositions varying by chlorine content. Each congener differs in chlorine number, arrangement, and molecular properties like lipophilicity and size, influencing their distribution between blood and milk and their toxicity [30]. Indicator congeners (e.g., PCB-153, PCB-138, PCB-180) and toxicologically relevant congeners (e.g., PCB-114, PCB-118, PCB-170) share similar molecular structures and metabolic pathways, largely due to the position of halogen substituents that confer structural rigidity and facilitate transfer to breast milk. Importantly, SHAP and SAGE findings reveal specific PCB congeners from the complex mixture as key contributors to PCB-170 prediction in human milk, highlighting their potential interactive effects. These joint effects are non-linear and concentration-dependent, with positive contributions at lower concentrations and inhibitory effects at higher levels, possibly reflecting saturation or competition mechanisms.

### 3.2. Limitations

Several limitations of the present study should be acknowledged. First, the analysis is based on a relatively limited dataset, which necessitates further validation across diverse populations and settings to enhance the robustness and generalizability of the findings. Second, while this study explores the behavior of PCB-170 in breast milk in relation to other congeners, maternal age, and parity, it does not account for other potentially influential variables, such as dietary patterns, occupational exposures, or physiological and lifestyle factors, that may significantly impact pollutant levels. Research shows that exposure to PCBs may increase the risk of pregnancy complications and pre-existing systemic diseases such as gestational diabetes, preeclampsia, thyroid disorders, and metabolic disorders. In turn, these health changes can affect lipid metabolism, hormone levels, and liver function, potentially influencing the distribution and excretion of PCBs into breast milk [38,39,40]. Third, although the integration of ensemble decision trees, metaheuristic optimization, and feature attribution methods (e.g., SHAP and SAGE) represents a novel methodological contribution to the field of human biomonitoring, the absence of comparative evaluation against established modeling techniques may limit interpretability and broader applicability. A more comprehensive understanding of the determinants of contaminant concentrations in breast milk requires access to richer datasets that incorporate a broader range of explanatory variables, particularly those reflecting maternal living conditions and detailed health profiles, including metabolic, physiological, and clinical parameters. The inclusion of such variables would substantially strengthen future modeling efforts and deepen insight into the complex interactions governing pollutant bioaccumulation and exposure risks.

In addition, the developed framework was applied to systematically analyzed, internally curated datasets, using rigorous validation to ensure reliability and interpretability. While consensus modeling is considered the best practice when multiple models or external datasets are available, its implementation depends on harmonized features, compatible outcomes, and large standardized datasets, conditions often unmet in studies involving highly specific biological samples. Future collaboration and participation in broader data-sharing initiatives may enable the use of consensus models to further strengthen the robustness and generalizability of findings in this field.

## 4. Conclusions

This study elucidates the complex co-occurrence and interaction patterns of the toxicologically significant PCB-170 with 23 other environmental pollutants in human breast milk, encompassing PCB congeners and organochlorine compounds from the HCH and DDT groups. Among these, hexa- and hepta-chlorinated congeners, specifically PCB-138, PCB-153, and PCB-180, along with *p*,*p’*-DDE, emerged as dominant predictors, while other POPs, child delivery, and maternal age showed negligible effects.

By leveraging the AI analytical framework, combining tree ensemble ML, metaheuristics, and explainable AI, this work supports previous findings on POP occurrence in breast milk and its associations with maternal factors such as age and parity, while also enhancing and deepening the understanding provided by traditional statistical methods through the capture of complex, nonlinear, and multidimensional interactions. SHAP-based interaction analysis revealed consistent, concentration-dependent joint effects among the most influential congener pairs, particularly PCB-180–PCB-153, PCB-138–PCB-180, and PCB-138–PCB-153. Positive interaction values in low concentration ranges suggest additive or synergistic effects, likely due to shared sources or similar environmental pathways. Conversely, negative interactions at higher levels point to biological saturation or competitive mechanisms, indicating that the combined influence of pollutants can attenuate rather than enhance predicted PCB-170 levels. Transitional interaction zones further highlight the nonlinearity of these relationships and suggest potential inter-individual variability in exposure dynamics or toxicokinetic responses.

These findings advance our understanding of POP chemical exposome by revealing individual PCB congeners and their combined effects as dominant contaminants in human breast milk. It underscores the need to revise current monitoring strategies with future investigations prioritizing the simultaneous assessment of multiple pollutants and the targeted inclusion of specific congeners, beyond those usually investigated, to better capture the complexity of maternal and child exposure to PCBs and OCPs. Despite current data limitations, the flexible and scalable AI framework shows strong potential for diverse applications in environmental modeling, supporting more accurate risk assessment through the identification and focusing on key congeners among numerous analyzed compounds.

## Figures and Tables

**Table 1:** Cluster-wise descriptive statistics incorporating SHAP-derived impact metrics and model prediction probabilities.

Cluster	Count	Percentage [%]	PCB170 [ng g^−1^]	Mean Impact	Mean Absolute Impact	Mean Normalized Impact [%]	Mean Probability
C-1	25	13.44	1.53	0.02	1.34	1.62	0
C0	13	6.99	1.07	−0.51	0.83	−33.26	0.74
C1	11	5.91	3.07	1.96	2.54	127.2	0.92
C2	11	5.91	7.99	5.97	6.04	387.58	0.7
C3	9	4.84	4.28	2.76	2.99	179.39	0.91
C4	9	4.84	0.64	−0.78	0.88	−50.37	0.95
C5	27	14.52	0.35	−1.17	1.17	−75.62	0.63
C6	17	9.14	0.25	−1.26	1.27	−81.70	0.68
C7	9	4.84	0.29	−1.25	1.26	−81.11	0.87
C8	34	18.28	0.56	−0.99	1.09	−64.00	0.7
C9	21	11.29	1.90	0.30	0.80	19.37	0.63

## Data Availability

The data presented in this study are available on request from the corresponding author as they are being used in other ongoing research.

## Supplementary Materials

The following supporting information can be downloaded at https://www.mdpi.com/article/10.3390/toxics13080631/s1. Table S1. Descriptive statistics for the study variables. Table S2. Best-performing model evaluation statistics. Figure S1. Variability and central tendencies of organochlorine compounds (PCB170, PCB180, PCB138, PCB153, PCB156, PCB118, and ppDDE) in human breast milk samples. Figure S2. Correlation matrix for the study variables. Figure S3. Best performed model evaluation report. Figure S4. Global importance of input features based on normalized SAGE values (values in % are annotated next to the respective bars). Figure S5. Correlation matrix of SHAP values of persistent organic pollutants and demographic predictors. Figure S6. SHAP interaction matrix.
