# Supplementary material for: Application of the AI-Based Framework for Analyzing the Dynamics of Persistent Organic Pollutants (POPs) in Human Breast Milk
Source: Toxics. 2025 Jul 27;13(8):631. doi: 10.3390/toxics13080631 (PMC12390066; doi:10.3390/toxics13080631)
Supplement: Supplementary file 1 [file toxics-13-00631-s001.zip › toxics-3728195-supplementary.pdf]

Application of the AI-based framework for analyzing the dynamics of persistent organic pollutants (POPs) in human breast milk

Gordana Jovanović[1], Timea Bezdan[2], Snježana Herceg Romanić[3], Marijana Matek Sarić[4], Martina Biošić[3], Gordana Mendaš[3], Andreja Stojić[1,2] and Mirjana Perišić[1,2]

[1]*Institute of Physics Belgrade, National Institute of the Republic of Serbia, Pregrevica 118, 11080 Belgrade Serbia; gordana.jovanovic@ipb.ac.rs (G.J.); mirjana.perisic@ipb.ac.rs (M.P.); andreja.stojić@ipb.ac.rs (A.S.)*
[2] *Singidunum University, Danijelova 32, 11000 Belgrade, Serbia; tbezdan@singidunum.ac.rs (T.B.)*
[3]*Institute for Medical Research and Occupational Health, Ksaverska cesta 2, PO Box 291, 10001 Zagreb, Croatia, sherceg@imi.hr (S.H.R.), gmendaš@imi.hr (G.M.), mbiosic@imi.hr (M.B.)*
[4]*Department of Health Studies, University of Zadar, Splitska 1, 23000 Zadar, Croatia, marsaric@unizd.hr (M.M.S.)*

*Corre□pondence: □herceg@imi.hr (S.H.R.)

Supplementary material

Table S1. Descriptive statistics for the study variables.

Variable	N	Mean	St. dev.	Min	25th perc	50th perc	75th perc	Max
beta-HCH	186	1.5423	2.3734	0.02	0.15	0.4563	2.0618	14.049
gamma-HCH	186	1.6033	3.508	0.02	0.15	0.4975	1.3577	30.8483
HCB	186	2.3486	3.2043	0	0.3907	1.099	2.9253	19.313
p,p'-DDE	186	7.2073	10.1916	0.01	1.78	4.4132	7.9925	77.7526
p,p'-DDD	186	2.1241	5.425	0.01	0.1	0.3	0.6921	49.484
p,p'-DDT	186	4.3478	9.3025	0.01	0.3	1.0683	5.1634	92.645
PCB-138	186	2.418	2.9162	0.01	0.3198	1.2983	3.4168	14.1166
PCB-153	186	4.9701	6.3623	0.01	1.0277	2.5782	5.9194	32.5428
PCB-180	186	2.3874	3.4263	0.0887	0.304	1.2043	2.5548	18.6998
PCB-105	186	0.4856	0.5988	0	0.25	0.25	0.5786	5.6885
PCB-118	186	0.4701	0.4897	0	0.25	0.25	0.6003	2.4414
PCB-123	186	0.2549	0.2459	0.02	0.25	0.25	0.25	2.6477
PCB-156	186	0.4589	0.5145	0.01	0.25	0.25	0.5621	2.8245
PCB-157	186	0.3546	0.5775	0	0.25	0.25	0.25	6.5268
PCB-189	186	0.2631	0.2719	0.0056	0.25	0.25	0.25	2.16
PCB-74	186	0.823	0.9847	0.0163	0.25	0.25	1.1631	5.9785

PCB-170	186	1.577	2.0791	0.01	0.25	0.7124	1.96	11.0146
Mothers Age	186	29.8763	5.6032	19	25	30	34	41
Birth	186	1.7688	0.7317	1	1	2	2	3

Table S2. Best-performing model evaluation statistics.

**Parameter**	**Value**
Mean absolute error (MAE)	0.4396
Mean squared error (MSE)	0.5408
Root mean squared error (RMSE)	0.7354
Mean absolute percentage error (MAPE)	0.3234
Explained variance	0.9123
Maximum error	2.0012
r^2	0.9125

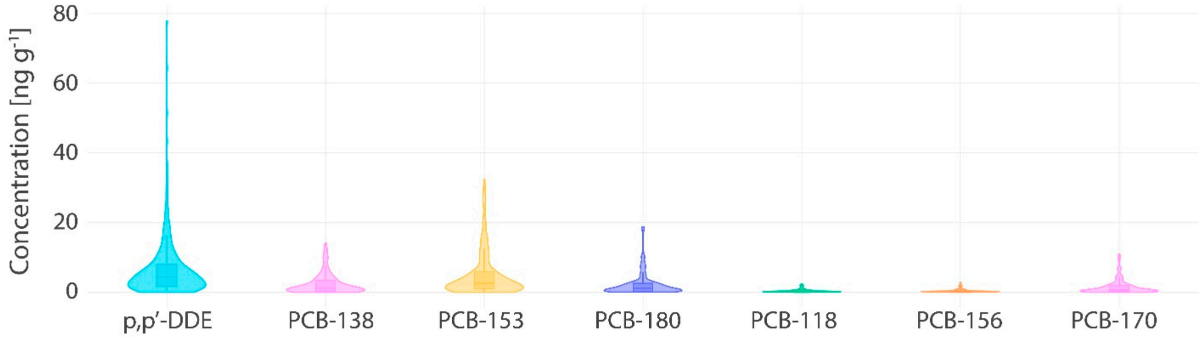

Figure S1. Variability and descriptive statistics~~central tendencies~~ of organochlorine compounds (PCB-170, PCB-180, PCB-138, PCB-153, PCB-156, PCB-118, and *p,p'*-DDE) in human breast milk samples.

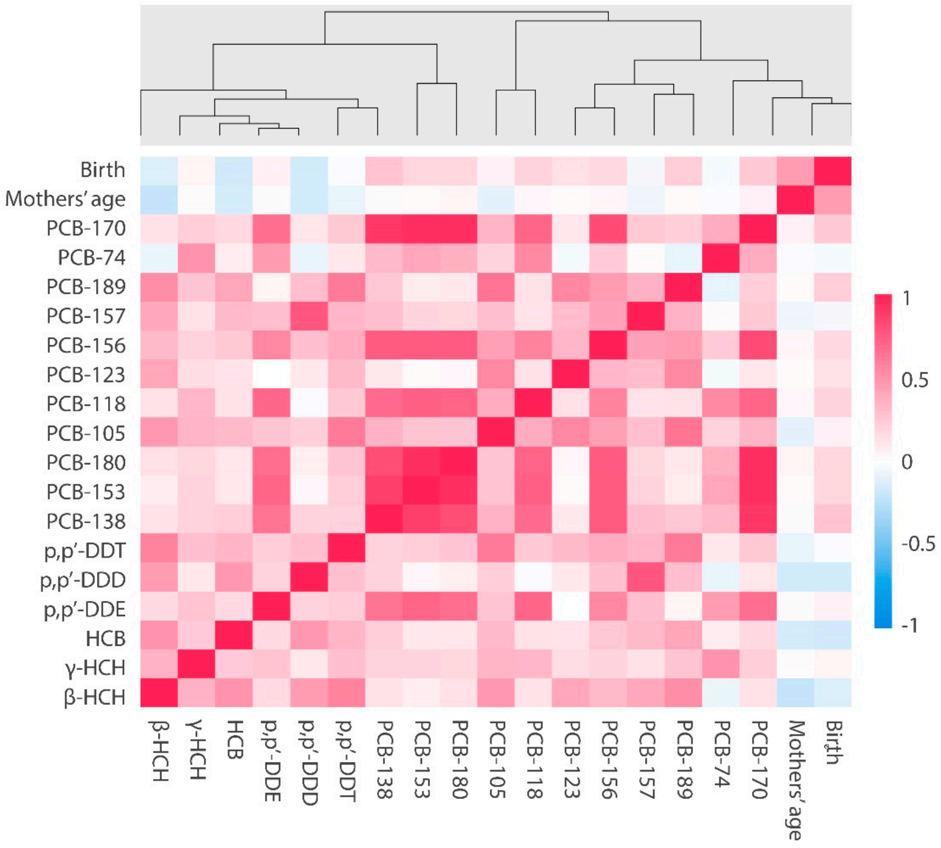

Figure S2. Correlation matrix for the study variables.

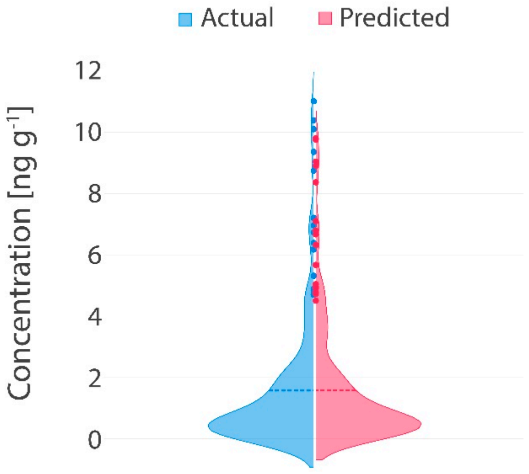

Figure S3. Actual/predicted instance distribution for the best performing model.~~Best performed model instance distribution.~~

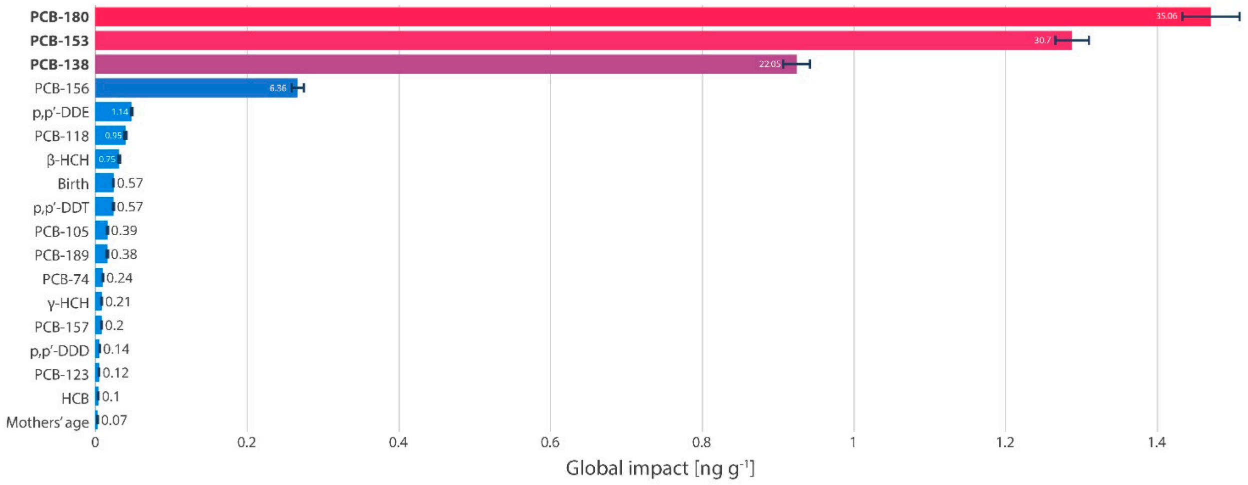

Figure S4. Global importance of input features based on normalized SAGE values (values in % are annotated next to the respective bars).

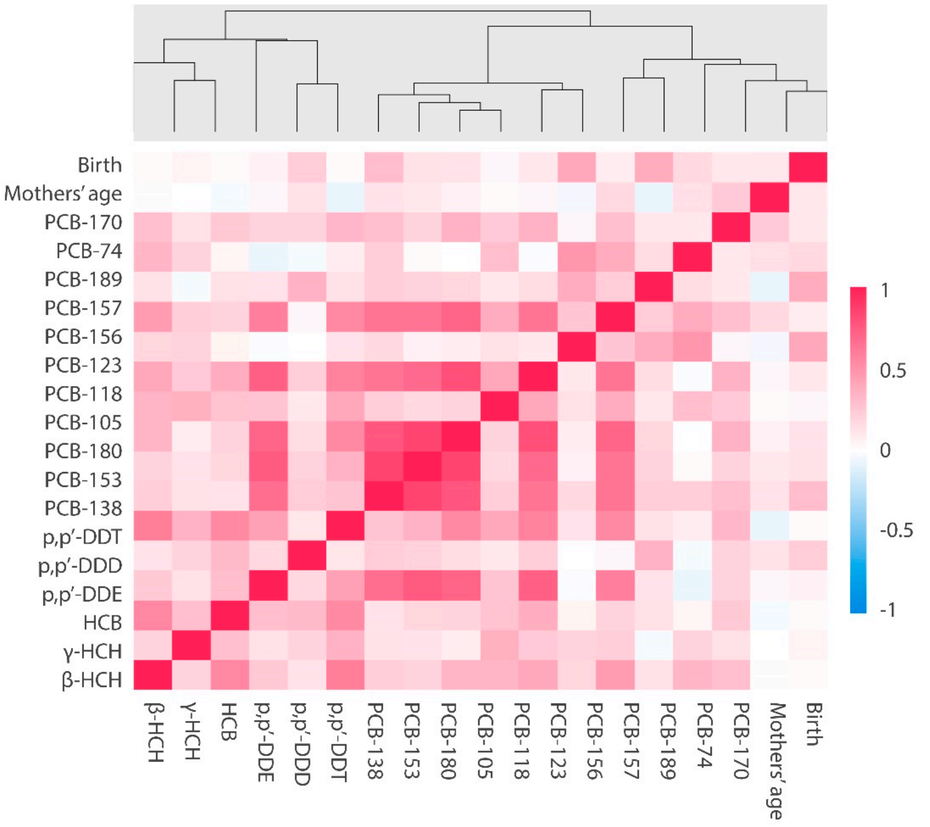

Figure S5. Correlation matrix of SHAP values of persistent organic pollutants and demographic predictors

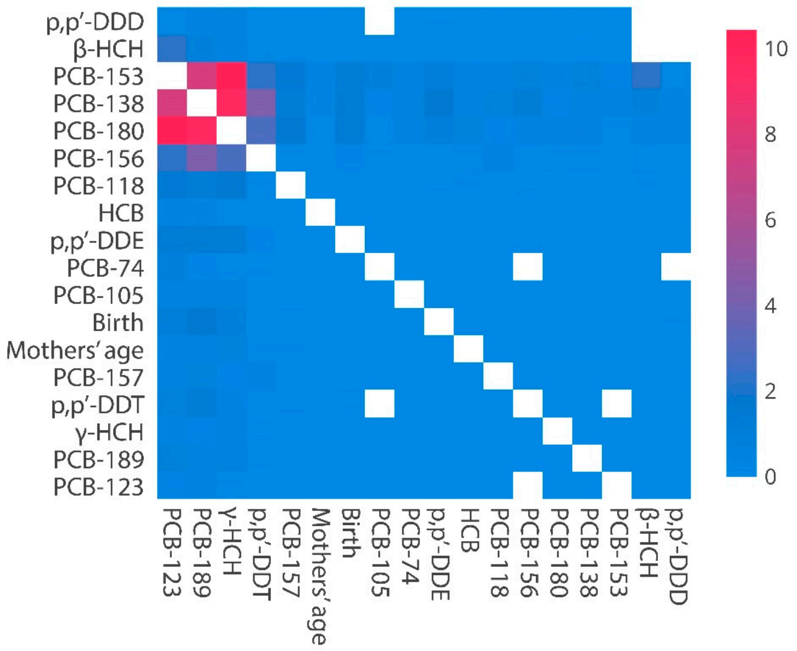

Figure S6. SHAP interaction matrix.
